# Simulating Timescale Dynamics of Network Traffic Using Homogeneous Modeling

**DOI:** 10.6028/jres.111.019

**Published:** 2006-06-01

**Authors:** Jian Yuan, Kevin L. Mills

**Affiliations:** Tsinghua University Beijing, China; National Institute of Standards and Technology, Gaithersburg, MD 20899

**Keywords:** correlation structure, modeling, network traffic, simulation, wavelet-based analysis

## Abstract

Simulating and understanding traffic dynamics in large networks are difficult and challenging due to the complexity of such networks and the limitations inherent in simulation modeling. Typically, simulation models used to study traffic dynamics include substantial detail representing protocol mechanisms across several layers of functionality. Such models must be restricted in space and time in order to be computationally tractable. We propose an alternative simulation approach that uses homogeneous modeling with an increased level of abstraction, in order to explore networks at larger space-time scales than otherwise feasible and to develop intuition and insight about the space-time dynamics of large networks. To illustrate the utility of our approach, we examine some current understandings of the timescale dynamics of network traffic, and we discuss some speculative results obtained with homogeneous modeling. Using a wavelet-based technique, we show correlation structures, and changes in correlation structures, of network traffic under variations in traffic sources, transport mechanisms, and network structure. Our simulation results justify further investigation of our approach, which might benefit from cross-verifications against more detailed simulation models.

## 1. Introduction

To design effective and efficient network protocols and to understand and solve performance problems arising in communication networks, designers and analysts require accurate models to describe network traffic. For these reasons, many researchers have studied, and continue to study, the characteristics of traffic entering and transiting the Internet. Measurements of local-area and wide-area Internet traffic [1–3] exhibit self-similarity and long-range dependence (LRD) at large timescales. This phenomenon is believed to arise as a consequence of heavy-tailed file sizes exchanged by network applications. For example, Park and colleagues [4] show how heavy-tailed distributions of file sizes at the application layer are preserved at the transport layer by the transmission-control protocol (TCP) and are then mapped to approximate heavy-tailed busy periods in the network layer. Empirical evidence also shows that intricate scaling dynamics arise over small timescales from the window-based, flow-control mechanism used by TCP [5]. Moreover, a recent study shows that over a finite range of timescales, independent from the timescales of application-layer traffic, TCP mechanisms can generate traffic with a similar correlation structure over similar timescales as found in measured Internet traffic [6,7]. Since the majority of Internet traffic is transported using TCP, there seems to be little doubt that many aspects of traffic dynamics relate directly or indirectly to TCP. Logically then, this understanding seems also to imply that traffic dynamics might change if another protocol supplants TCP as the dominant transport protocol in the Internet. While the transport protocol might influence substantially the characteristics of network traffic, some additional factors, such as network scale and load, not yet investigated thoroughly, could also contribute to the spatiotemporal dynamics in network traffic. These important questions surrounding the factors that influence the dynamics of network traffic remain subject to research and debate.

Providing a comprehensive explanation of the dynamic characteristics of network traffic presents a difficult challenge because the Internet is complex, and network models encounter difficulty representing, in a computationally tractable form, what could prove to be some of the most significant factors. For example, many simulations, used to illustrate some points regarding network behavior, are constrained to a small number of competing traffic sources and a single congested link [4,5,9,10], whereas, in a large network with realistic traffic, connections adapt to multiple congestion bottlenecks anywhere along the end-to-end path between sender and receiver. Small, simple simulations cannot capture spatiotemporal correlations of congestion, loss rate, and cross traffic, and thus variations of available bandwidth over large timescales, which occur naturally in a large distributed network. Such time-varying conditions are essential to reveal the timescale dynamics of network traffic, which depends on interwoven couplings and delayed feedback. In this paper, we attempt to simulate time-varying behavior of network traffic by representing complex details of a network in a specific modeling framework.

### 1.1 Motivation

Increasingly, networking measurement and modeling research seems to focus on issues related to scale, yet various researchers can conceive the meaning of scale quite differently. Some researchers use scale to denote system size, while others use scale in relation to the precision of description or observation. While the former conception conveys a sense of space, the latter conception encompasses two meanings of time: fineness, which suggests the operational time-step of the evolving model in simulation, and granularity, which indicates the observation interval of the system in evolution. We suspect that relationships among these meanings of scale wield significant influence over the effectiveness of particular modeling methodologies for specific aims. For example, it appears difficult to replicate the dynamics of real network traffic over large timescales by using simulations of small size [10,14,15].

To study large-scale characteristics and long-term phenomena, and to analyze a system as a whole, seems then to require simulating networks of large size. However, the need for both representational effectiveness and computational convenience present network modelers with two conflicting factors: simulation size (i.e., the number of elements) and level of description (i.e., the detail represented within each element). Simulation of large networks is difficult due to sheer size. Moreover, size is compounded with all kinds of configuration parameters, such as topology, link capacity, and data flow characteristics. The spatial heterogeneity of large networks, including both static factors (e.g., network capacity assignment) and dynamic factors (e.g., traffic load variations), challenges network modelers wishing to simulate large networks, because the larger the model size and the finer the model detail, the more voluminous the parameter space of the model. For example, some researchers observe the difficulty of selecting meaningful topologies, especially in the case of large network size [11]. Lacking a “typical” network topology, even if we limit our investigation to a single measure, we face a huge parameter space of possible topologies that might be searched.

In addition to the compounding of size with representational detail, temporal detail also combines with size to further increase the degree of modeling difficulty. Generally, the real dynamics of complex systems rely on basic interactions at the finest timescale. Since the behaviors of a system at different timescales are related, modeling descriptions should include these relationships. Usually, detailed simulations do include per-packet modeling but only for small networks (on the order of 10 to 10^2^ nodes). While simulation of large networks may pay more attention to large timescale behaviors, such simulators must model the full-duplex “ripple effect” of network flow control in order to represent crucial dynamics at the per-packet level, which have global effect on network congestion [12]. This implies that a good, detailed simulation of a large network requires almost impractical execution time, not only to overcome the long transient startup period, but also to collect sufficient data to support reliable analysis of long-term correlation. Even if parallel simulation techniques can reduce the total execution time to some extent [13], the time needed to vary and search the huge space of configuration parameters to generate comparative results is intractable for sufficiently detailed models of large networks on the order of 10^5^ to 10^6^ nodes. It seems inevitable, then, that we must make some simplifications and abstractions to model network dynamics at large scale.

Abstracting an appropriate model depends very much on objectives. When studying the dynamic behavior of network traffic at multiple timescales, there seems little doubt that the natural coupling among multiple timescales is crucial, and therefore must be represented in the model. That is, the model must meet the challenge of representing both large size and packet-level detail. On the other hand, there seems to be little idea about the effects of the spatial factors (e.g., topology, link capacity assignment, and host distribution) on timescale dynamics. While heterogeneity and hierarchy are sometimes emphasized in network simulations [10,11], the spatiotemporal effects (e.g., correlation at large time intervals) of the geographically distributed structure of large networks (which can exhibit multiple congestion points) seems to be largely ignored in investigations of the timescale dynamics of network traffic.

One project aims to model the global Internet using the scalable simulation framework (SSF), which was developed to construct and execute very large, parallel models [14]. SSFnet (an extension of SSF) permits one to analyze the detailed behavior of large, multi-domain, multi-protocol Internet models. The SSF developers claim that SSFnet can execute detailed simulations of complex network topologies with a million or more concurrent transport flows. Unfortunately, SSFnet currently ignores the details of link-level transmission beyond gross characterizations of bandwidth and transmission delays [14]. This half-duplex strategy, which can significantly reduce simulation time, fails to produce the delicate, but crucial, “ripple effect” throughout a network. Without this “ripple effect”, simulated network traffic can possibly differ from the global effect at large timescales [15]. Clearly, representation of spatiotemporal detail challenges the current state-of-the-art when simulating large networks over long timescales.

### 1.2 Modeling Methodology

Some studies in physics use a cellular automaton (CA) [16]. A CA is a discrete dynamic system composed of a set of cells arranged in a regular, one or multidimensional spatial lattice. CA studies suggest that homogeneous models can provide significant insight into the behavior of real-world phenomena, even though the real world is rarely homogeneous. The goal of CA models is usually to illuminate the irregular dynamics of a system through a regular model, and to build an understanding of essential features that might hold for a real system corresponding to the model. Physicists explore CA models because they can sometimes provide significant insights at less computational cost than would prove feasible with more detailed models. We suspect that the homogeneous modeling approach adopted in this paper may exhibit realistic traffic dynamics over large timescales because the increased level of abstraction within a CA model allows us to include essential spatiotemporal details, while retaining computational tractability at large size.

In structuring our model, we used several assumptions that we believe to be reasonable; specifically, the model should: (1) describe a set of interconnected domains, (2) characterize user behavior, and (3) simulate adaptive packet traffic, under a transport protocol, at a scale sufficient to accommodate spatial correlations that might emerge in a real network. To achieve a sufficiently large model with well-understood parameters, we resort to a methodology of simplification: homogenization, which lessens the combined effects of size and topological detail. Our methodology uses homogeneous modeling of a regular, multi-domain, two-tiered topology of nodes and links, but includes multiple layers of protocol. This homogenized model includes fewer processing steps than more detailed discrete-event simulation models that require substantial computation, much of which may be devoted to modeling behaviors irrelevant to the network dynamics being investigated. Employing a homogeneous model enables us to somewhat reduce the computational burden of simulation, allowing us to simulate larger networks for longer durations.

We study the traffic dynamics arising from interactions among user behavior (the application layer), transmission dynamics (the transport layer), and network structure (link topology and capacity) under a homogeneous modeling framework. First, we verify our findings against the current understanding of timescale dynamics in network traffic. Subsequently, we consider how traffic dynamics can change with changes in user behavior, transport protocol, or network structure. Though we confront a large, complex system whose behavior defies simple explanation, our homogeneous modeling offers a clear-cut framework to identify if a measure of interest is sensitive to each changed variable. Before providing the details of our method, we briefly outline the significant factors in our investigation, overview our analysis approach, and give a summary of our results.

### 1.3 User Behavior

We model user behavior as ON/OFF sources, considering both heavy-tailed and exponential distributions. Aggregating heavy-tailed ON and/or OFF periods for individual traffic sources can lead to long-range dependence (LRD) in the aggregated process [17]. For this reason, we use the Pareto distribution to model heavy-tailed ON/OFF periods. To identify factors arising independent of heavy-tailed traffic sources, we use the exponential distribution to represent user behavior without heavy-tailed characteristics. We also consider effects arising from varying the mean value of ON/OFF periods.

### 1.4 Transmission Dynamics

We model transmission dynamics arising from two different congestion-control mechanisms. One mechanism models TCP, the dominant transport protocol used to convey traffic in the current Internet. By probing for spare capacity and reacting to path congestion, TCP produces rapidly varying transmission rates. TCP has a strong influence on the behavior of traffic at small timescales, but also seems to have more sensitivity [18] to fluctuations over large timescales, when compared to some more slowly responding congestion-control mechanisms [19]. Slowly responding congestion-control mechanisms appear better suited than TCP for selected applications, such as best-effort, unicast streaming video and audio. To represent this second class of congestion-control mechanism, we model the TCP Friendly Rate Control Protocol (TFRC), which uses an equation-based, congestion-control mechanism [20]. TFRC [21] is one of several congestion-control mechanisms proposed for use in the future Internet. By modeling two different congestion-control protocols, we investigate the influence of control mechanisms on the timescale dynamics of network traffic. We can also explore how relationships between user behavior and congestion-control mechanism might alter timescale dynamics.

### 1.5 Network Structure

For the network structure, we use a mesh topology that comprises numbers of interconnected domains, each of which has two tiers: an upper tier for routers and a lower tier for hosts. In the lower tier, a variable number of hosts can attach to each router. At the upper tier, routers connect to each other in a regular way to form the whole network. In this paper, while considering changes in both the number of hosts per router and the capacities of links connecting routers, we pay particular attention to the effects of network size—a property of the upper tier—on the dynamics of network traffic at timescales greater than round-trip times. At these timescales in a large network, the traffic dynamics might be influenced largely by spatiotemporal adaptation to congestion arising from cross traffic in routers.

### 1.6 Analysis Approach

Our analysis of the timescale dynamics of network traffic adopts the wavelet-based technique proposed by Abry and Veitch [22]. This technique provides a natural and effective means to investigate the correlation structure of network traffic, and to identify and extract regular patterns in a way that cannot be easily accomplished with Fourier-based techniques [5–8]. Our results are qualitative in nature rather than quantitative. For example, we examine ranges of timescales, we identify regions where a correlation structure holds, and we detect changes in correlation structure. Usually, achieving a reliable long-correlation analysis using wavelets depends on obtaining sufficient data, which implies long simulation time, especially for a large network.

### 1.7 Results Summary

With our model, we indeed reproduce the small-scale, middle-scale, and large-scale dynamics of network traffic, which have been explained extensively by network researchers [4–8]. First, we verify that high variability in file sizes can result in a strong correlation structure over a wide range of timescales, and low variability yields linear structure over only a limited range. Second, we verify that TCP plays an important role in traffic characteristics from fine-scale to large-scale. However, we also show that while both TCP and TFRC can produce strong correlation over a limited range of timescales themselves, their influence on the correlation structure can differ, especially at large timescales, where TFRC sometimes does not preserve the LRD structure induced by high user variability. Third, we show that offered traffic and shared network capacity combine to act as traffic-shapers, strengthening and loosening correlation structure, which can be offset by congestion-control mechanisms. Finally, we also illustrate a simulation result that shows a similar correlation structure to that seen for measured Internet traffic. This result suggests that a LRD correlation structure might be expected to arise in very large networks, even without high user variability. We provide a plausible explanation for our result, based on the current understanding of the timescale dynamics of network traffic.

### 1.8 Organization

The remainder of the paper is structured as three sections. Section 2 discusses our modeling and analysis approaches. We describe our representation of ON/OFF traffic sources, congestion-control algorithms, network structure, and routing. We also outline the wavelet-based analysis method. In Sec. 3, we delineate our experiments and show our simulation results. We distinguish among the effects of user behavior, transmission dynamics, and network structure. We present concluding remarks in Sec. 4.

## 2. Method

Simulating and understanding behaviors in a large network at large timescales present difficult challenges. However, if we restrict our simulation appropriately, then we can perhaps find a tractable approach that helps us to develop intuition and insight. Earlier results, reported elsewhere [15], encouraged us to continue our attempt at modeling large networks using a homogenous cellular automaton (CA). In the work reported here, we improve almost all aspects of our earlier work. We adopt a homogeneous modeling approach that enables us to simulate a large network, while varying selected factors: link capacity, traffic generation, transport mechanism, and network size. Using such a simulation, we can observe changes in the correlation structure of network traffic when varying each factor, one by one.

### 2.1 Modeling

To provide a holistic view of network traffic, a model should encompass the variability and complexity inherent in a large network, including the effects of network-host interactions and the effects of protocol regimes and network controls. Though we restrict our attention to comparative aspects of the timescale dynamics of network traffic, our model must still capture some important details of ON/OFF sources, congestion-control algorithms, network structure, and routing.

#### 2.1.1 ON/OFF Sources

In this paper, each source models traffic generation as an ON/OFF process, which alternates between wake and sleep periods. When awake a source may send, subject to any restrictions imposed by the congestion-control algorithms, one packet at each time-step to the source’s first-hop router. The packet will be placed at the end of the router’s queue. At the beginning of each “ON” period, a source randomly selects (uniform distribution) another routing domain (with an available destination host) as its sink. Each packet generated by the source during the same “ON” period has the same destination address. When sleeping, the source does not generate new packets at each time-step. ON/OFF sources provide a convenient model of user behavior.

We can modulate the frequency and duration of ON/OFF periods by selecting arrival and departure times according to various statistical distributions. Here, we use one of two distributions. Most empirical measurements on the Internet observe a heavy-tailed distribution of transferred file sizes. Some researchers believe long-range dependence arises in the Internet from the high user variability represented by such heavy-tailed distributions. To investigate this belief, we need a distribution to represent such user behavior. We also need another distribution that represents lower user variability. In this latter case, we represent the wake and sleep period durations as exponential processes with parameters *λ*_on_ and *λ*_off_.

To model high user variability, we represent the wake and sleep period durations using the Pareto distribution, which is frequently used to model the heavy-tailed characteristic of Internet file transfers. The Pareto distribution function has the form

$$P\left[X\leq x\right]=1-{\left(k/x\right)}^{\alpha },\;0<k\leq x,$$ (1)

where 0 < *α* < 2 is the shape parameter. The mean is given by *α k*/(*α* − 1) (for *α* > 1). Here, we use *α* = 1.2.

In this paper, we sometimes mix the Pareto and exponential distributions, using a Pareto “ON” period, and an exponential “OFF” period with *λ*_off_. To keep the same average “ON” duration for both distributions, let

$$k=\left(\alpha -1\right)\lambda_{\text{on}}/\alpha .$$ (2)

#### 2.1.2 Congestion-Control Algorithms

To achieve traffic dynamics across all timescales, our model consists of a parallel system at the packet level, where packets transit along connections between source-destination pairs. Every connection operates full duplex under one of two traffic-control regimes: TCP or TCP friendly rate control (TFRC) [21].

Modern TCP implementations contain four intertwined algorithms: slow start, congestion avoidance, fast retransmit, and fast recovery. In this paper, we model these details of TCP, in which fast retransmit will be performed to reduce the congestion window to half the current window size after receiving one, instead of three, duplicate acknowledgments for the same packet. While packets can be lost in our model, all packets on a connection take the same route, so no packet reordering occurs.

TFRC, relative to TCP, has a more smoothly varying transmission rate. The corresponding cost is a slower response to transient changes in congestion or to sudden increases in available bandwidth [19]. TFRC uses a receiver-driven mechanism, where the receiver calculates congestion-control information—i.e., the loss rate—and feeds back to the sender. The sender also uses these feedback messages to measure the round-trip time (RTT). The sender inputs the loss rate and RTT into a throughput equation, which yields an acceptable transmission rate. Then, the sender in our model adjusts its interval between packet transmissions to match the acceptable rate.

Whether used in TCP or TFRC, each packet in our model carries several pieces of information: source address (router and host), destination address (router and host), creation time, and sequence number. In addition, the sender and the receiver on each connection maintain state information, and exchange information via packets. In particular, for TCP the receiver maintains the expected sequence number to identify if a packet is lost, and to inform the sender when a packet drop occurs. For TFRC, the receiver maintains estimates of the loss rate and the packets-received rate, and records the timestamp of the last packet received. The receiver periodically sends this information, along with an estimated RTT, to the sender.

#### 2.1.3 Network Structure

**Fig. 1:**
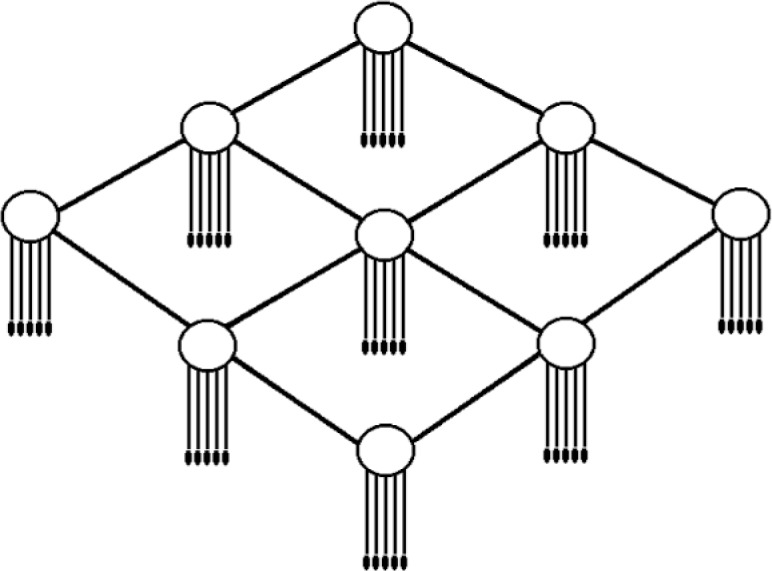
The network structure.

The topology of our model comprises numbers of interconnected domains, each of which has two tiers: an upper tier for routers and a lower tier for hosts, as shown in Fig. 1. In the upper tier, routers connect to each other in a regular way to form a mesh-like (grid) network. Each domain includes an equal number of sources (*n*_s_) attached to its router. There are also some other hosts attached to the router to take the role of receivers. When a source initiates a connection (ON period), a destination routing domain is chosen randomly, and an available receiving host is assigned. The destination router must differ from the source router. A receiver is released when the source ends the connection. We limit the number of receivers for each routing domain to double the number of sources (2*n*_s_). If a source selects a destination routing domain where all receivers are occupied, then another routing domain is selected randomly.

**Fig. 2:**
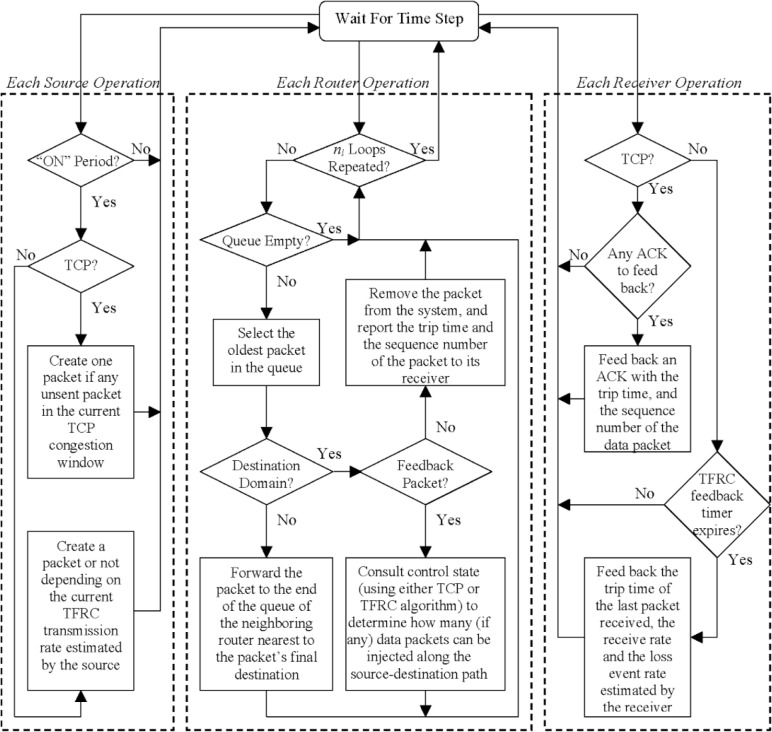
General parallel operations of sources, routers, and receivers at each time step (note that, TCP is Transmission Control Protocol, TFRC is TCP Friendly Rate Control, and ACK is acknowledgment).

Our model operates at the packet level. To store and forward packets traveling between source-destination pairs, each router maintains a queue of limited length (50 packets in this paper), where arriving packets are stored until they can be processed: first-in, first-out. In every discrete simulation time-step, if a source is in the ON period, the source can create one packet under the control of the transport protocol, and forward it to the buffer of its directly attached router. However, each router can forward multiple packets (*n*_l_) during each time-step. This forms a natural difference between two types of links in the two tiers. That is, the link capacity in the host tier is one packet-per-time-step (ppts), while the link capacity in the routing tier is *n*_l_ ppts. Figure 2 provides a schematic diagram of the parallel operations of sources, routers, and receivers at each time-step.

In this paper, we consider three parameters of the network structure: the number of sources (*n*_s_), the link capacity of the routing tier (*n*_l_), and the network size (*L*^2^), where *L* is the number of routers along one side of the grid. In Fig. 1, for example, *L* = 3 and *n*_s_ = 5; thus the network contains 9 routers and up to 45 simultaneously active connections. (Fig. 1 omits receivers.)

#### 2.1.4 Routing

In this paper, each source-destination pair has a constant, shortest path. To increase the size of network that we can model, instead of maintaining a forwarding table for each router, we compute a routing for each packet. To select the proper next-hop along which to forward a packet, the forwarding router computes the distance from each of its four neighboring routers to the packet’s destination router. Given the regular grid topology of our model, distance calculations can be performed easily. To determine the distance between routers, following Fuks [23], we use a metric defined for models with a periodic boundary condition, without which an unrealistic pattern of congestion may occur within the center of this regular network. Where multiple neighboring routers prove equidistant from the destination (at most two choices in our model), we consistently choose the left direction, which provides a constant path for all packets on a connection. Then the packet is forwarded to the next-hop router. Our routing technique leads to a constant, shortest path for each source-destination pair in one direction, and a different path for the reverse direction.

### 2.2 Wavelet-Based Analysis Technique

Characterizing network traffic requires knowledge about the statistical properties of packet arrivals over various timescales. Empirical network traffic exhibits LRD at large timescales, as manifested by slowly decaying autocorrelations. LRD usually represents one of several equivalent ways to describe second-order self-similarity [17], but correlation structure is a more general construct. Correlation structure can be observed through the autocorrelation function, or its Fourier transform—i.e., the power-spectral density. As indicated by Feldman and colleagues [7], the periodogram method exhibits possible pitfalls. On the other hand, a wavelet-based technique [22], which is frequently used to analyze long-range dependent data and to estimate the associated Hurst parameter, provides a natural and effective tool to reveal the correlation structure of network traffic across a wide range of timescales. Because the wavelet transform divides data into different frequency components and analyzes each component with a resolution matched to its scale, the coefficients of wavelet decomposition can be used directly to study the scale (or frequency) dependent properties of data. The discrete wavelet transform represents a time series *X* of size *N* at a scaling level *j* by a set of wavelet coefficients *d_X_*(*j*, *k*), *k* = 1, 2, …, *N_j_*, where *N* = 2^−^*^j^N*. The coefficient |*d_X_*(*j*, *k*)|^2^ measures the amount of energy in *X* about the time 2*^j^k* and about the frequency 2^−^*^j^f*_0_, where *f*_0_ is determined by the sampling rate of the time series and the choice of the analyzing wavelet. Average energy at scale *j* (where scale *j* is referred to as an octave) is the average of the sum of the squared wavelet coefficients |*d_X_*(*j*, *k*)|^2^; i.e.,

$$E_{j}=\frac{1}{N_{j}}\sum_{k}{\left|d_{X}\left(j,k\right)\right|}^{2}‎$$ (3)

*E_j_* is really an estimate of the power-spectral density about the frequency 2^−^*^j^f*_0_ [7]. We plot log_2_ *E_j_* as a function of scale *j*, from finest to coarsest scales, and investigate the structure in the energy-scale plot.

## 3. Simulations

Other researchers [3–8] have investigated some aspects of user and network behavior that contribute to different characteristics in the dynamics of network traffic. We attempt to check the results obtained with our model against some of the existing results. We also study the effects of varying traffic sources, transport mechanisms, and network structure. Some of our results should prompt further exploration of the nature of network traffic, and perhaps inspire new ideas for engineering networks.

### 3.1 Effect of Application Level

Using an abstract ON/OFF model to mimic user behavior (a property of the application level), we consider the effect of three parameters: mean values of ON/OFF durations (*λ*_on_ and *λ*_off_), and, when using the Pareto distribution for the “ON” period, the shape parameter *α*. We first investigate the following network configuration: network size *L* = 3, number of sources *n*_s_ = 10, and link capacity *n*_l_ = 5. We use TCP as the transport level. The application level comprises exponentially distributed ON/OFF periods with *λ*_on_ = 200 and *λ*_off_ = 2000.

**Fig. 3:**
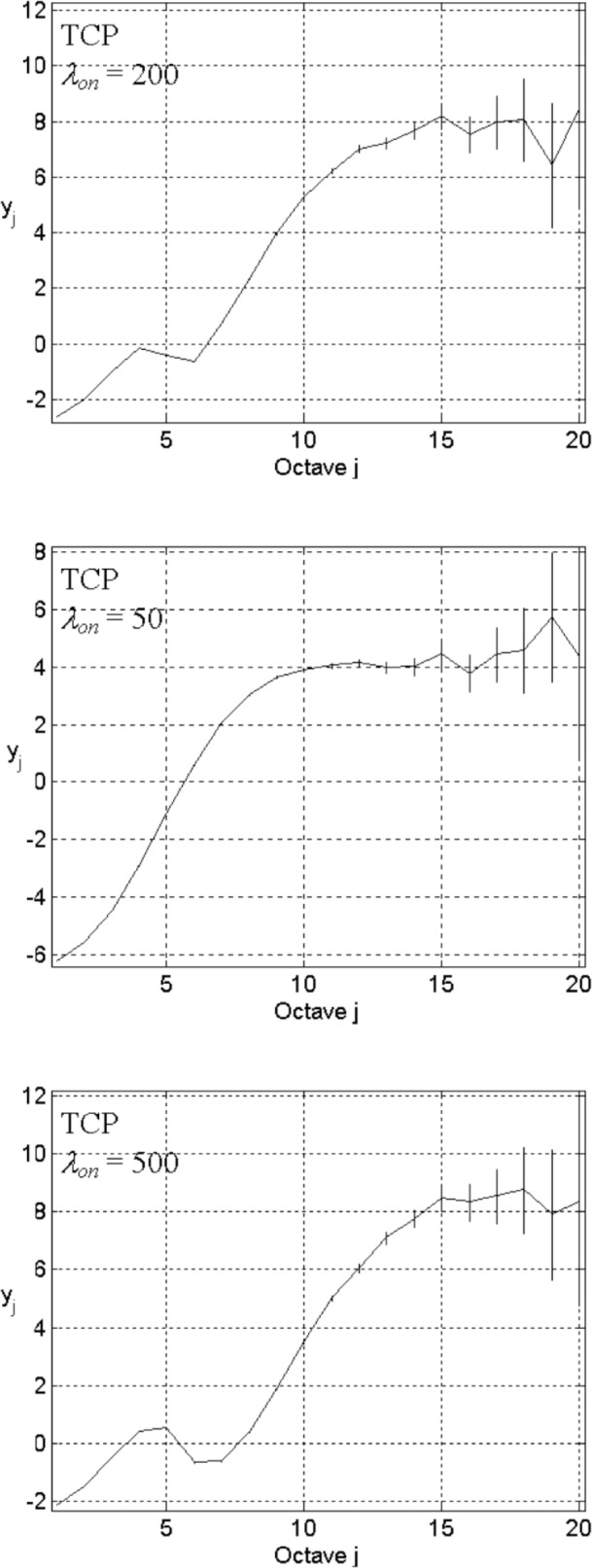
Energy-scale plots for *L* = 3, *n*_s_ = 10, *n*_l_ = 5, TCP in transport level, the exponential distribution ON/OFF with *λ*_on_ = 200 (top), 50 (middle), 500 (bottom), and *λ*_off_ = 2000.

Starting with a random initial condition, after discarding a transient period of 10^4^ time-steps, we analyze the traffic on one link between routers in one direction. We plot the resulting correlation structure as *y_j_* = log_2_ *E_j_* vs *j* (the top plot in Fig. 3). In this paper, we record enough data to yield an energy-scale plot that spans 20 octaves. Note that the finest scale description of traffic dynamics depends on the selection of *n*_l_. We focus solely on the large timescale features, checking for a more or less linear relationship.

In the corresponding plot of Fig. 3, we observe a strong correlation structure that spans around 5 octaves. The curve departs from linearity at the medium-to-small timescale (*j* < 6), and becomes flat after *j* ≥ 11. The linear part of the curve implies that the autocorrelation function decays according to a power law within a limited range of timescales. The flat part of the curve indicates that the autocorrelation function decays exponentially. Obviously the correlation structure is not consistent with LRD. The correlation structure is however consistent with similar results reported by Feldman and colleagues [5,7].

To investigate interaction between network congestion and offered traffic, absent high user variability, we keep all parameters fixed except for *λ*_on_, where we use 50 and 500, shown in the middle and bottom energy-scale plots of Fig. 3. We can see that changing *λ*_on_ alters the correlation structure. Specifically, as *λ*_on_ increases, the low-frequency energy (right portion of the energy-scale plot) tends to increase, and the linear part extends over a wider range of timescales. A similar effect also appears when changing *λ*_off_.

**Fig. 4:**
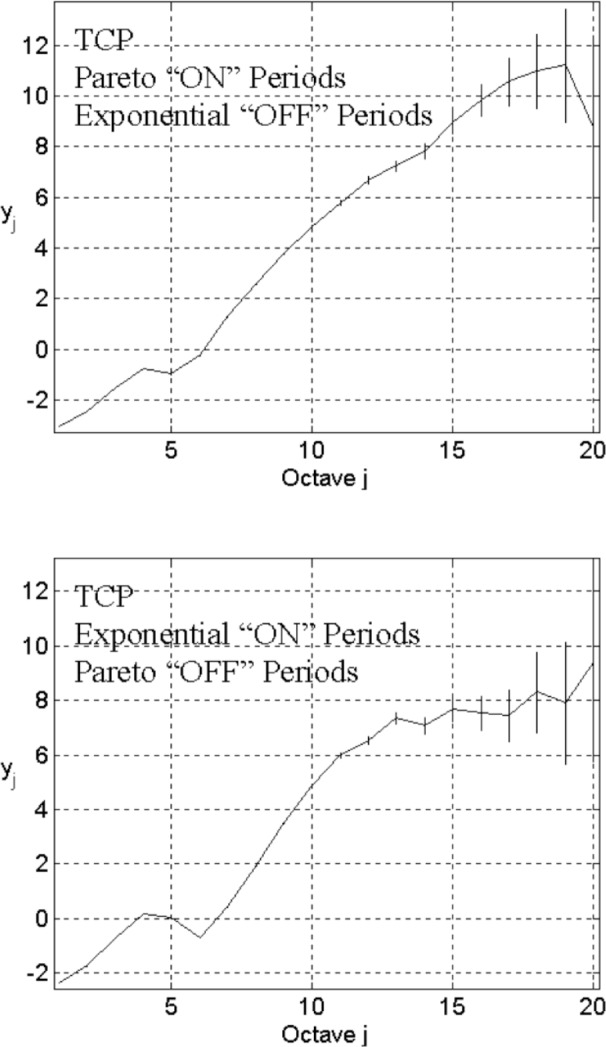
Energy-scale plots for *L* = 3, *n*_s_ = 10, *n*_l_ = 5, *λ*_on_ = 200, and *λ*_off_ = 2000, TCP in transport level, and the Pareto distribution “ON” (*α* = 1.2) with the exponential “OFF” (top), or the Pareto distribution “OFF” (*α* = 1.2) with the exponential “ON” (bottom).

To consider the effect of high user variability, we keep the same network configuration except for the “ON” period, where we use the Pareto distribution (*λ*_on_ = 200, *α* = 1.2, and *λ*_off_ = 2000). The top plot in Fig. 4 gives the corresponding energy-scale plot, which verifies previous results [3–5,8] that heavy-tailed file sizes lead to LRD over large timescales. Varying *λ*_on_ and *λ*_off_, we find this characteristic is robust. However, we cannot find this characteristic if we use a Pareto distribution to model heavy-tailed idle time. For example, keeping the same parameters, except using exponential “ON” periods and Pareto “OFF” periods (*λ*_on_ = 200, and *λ*_off_ = 2000, *α* = 1.2), the energy-scale plot in the bottom of Fig. 4 exhibits a flattening structure in its low-frequency part.

In summary, high variability in file sizes can result in a strong correlation structure over a wide range of timescales, while low variability yields linear structure over only a limited range. The correlation structures arise from many connections interacting under dynamic conditions, which can be shaped by offered load. Others [6,7] have suggested that two mechanisms inside TCP—exponential timeout back-off and congestion avoidance—contribute to the correlation structure. Will the same correlation structure arise when transporting data using a congestion-control mechanism without one or both of these TCP mechanisms? We investigate this question next.

### 3.2 Effect of Transport Level

**Fig. 5:**
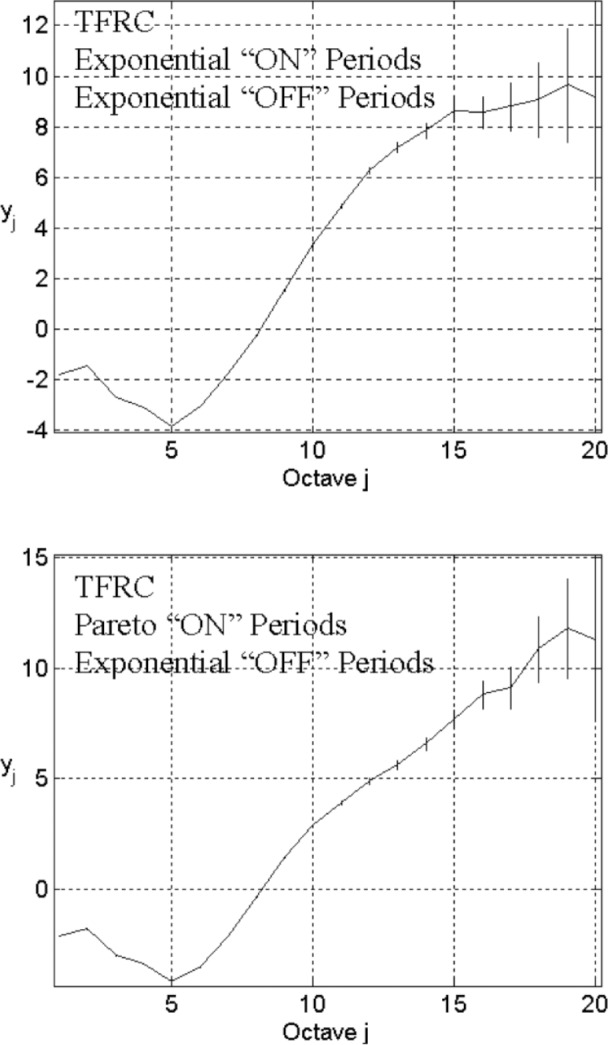
Energy-scale plots for *L* = 3, *n*_s_ = 10, *n*_l_ = 5, *λ*_on_ = 200, and *λ*_off_ = 2000, TFRC in transport level, and the exponential distribution ON/OFF (top), or the Pareto distribution “ON” (*α* = 1.2) with the exponential “OFF” (bottom).

We set up the network configuration with the same parameters used to obtain the top plot in Fig. 3, except that TFRC replaces TCP. The energy-scale plot appears as the top graph in Fig. 5. We find that the correlation structure at small timescales differs from the case of TCP (top plot in Fig. 3). However, similar to TCP, a strong correlation structure spans a limited range of octaves (*j* = 6 to 12). We also observed (not shown here) that varying “ON” and “OFF” periods (i.e., changes in offered traffic) has similar effects on the correlation structure, whether using TCP or TFRC. So it seems that the limited strong correlation structure does not rely on particular transport mechanisms. Congestion feedback algorithms, other than those used in TCP, may also yield a limited strong correlation structure as a result of interactions among many connections.

What about the effects of high user variability? Will heavy-tailed file sizes lead to LRD under TFRC? Other researchers [4,9] believe that, under TCP or flow-controlled UDP, LRD of aggregate traffic will appear as long as connection durations or object sizes being transported are heavy-tailed. In other words, they believe that LRD in aggregate traffic is insensitive to details in the protocol stack or the network configuration. Despite such beliefs, we have not found any research that investigates this question using any transport protocol other than TCP or an open-loop flow-controlled unreliable transport protocol, used by Park and colleagues [9]. We investigate the question with TFRC.

Keeping the same network configuration (see Fig. 5), we substitute the Pareto distribution “ON” (*λ*_on_ = 200, *α* = 1.2, and *λ*_off_ = 2000) in place of exponential “ON”. From the correlation structure in the corresponding energy-scale plot, which appears as the bottom graph in Fig. 5, heavy-tailed file sizes appear to give rise to LRD over large timescales under TFRC.

**Fig. 6:**
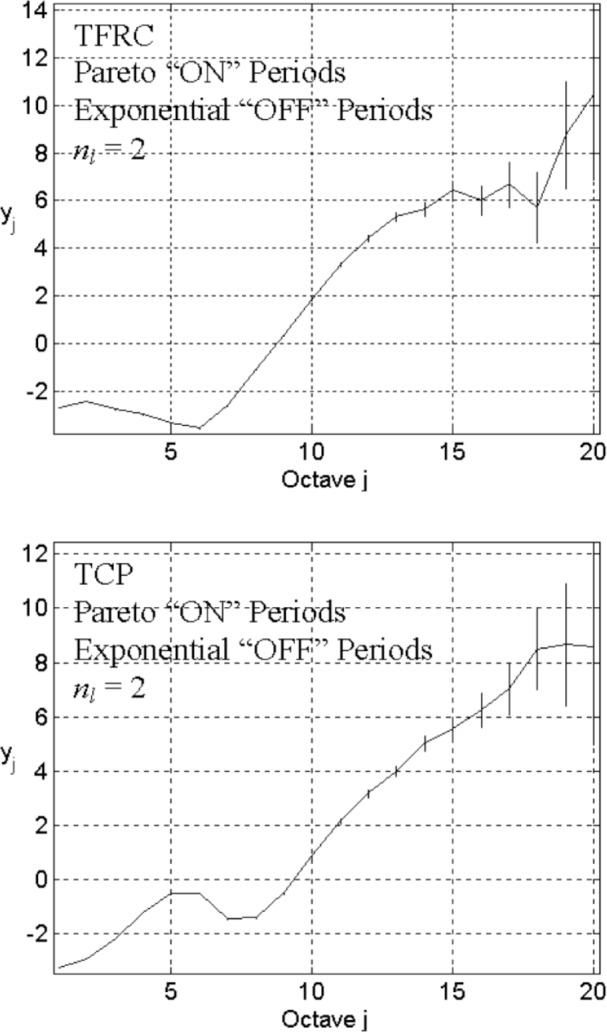
Energy-scale plots for *L* = 3, *n*_s_ = 10, *n*_l_ = 2, *λ*_on_ = 200, and *λ*_off_ = 2000, the Pareto distribution “ON” (*α* = 1.2) with the exponential “OFF”, and TFRC (top), or TCP (bottom) in transport level.

In the next experiment, we change only one parameter, reducing the link capacity to *n*_l_ = 2. The corresponding energy-scale plot (top graph in Fig. 6) does not exhibit the expected correlation structure. For TFRC, shrinking link capacity destroys the LRD structure induced by heavy-tailed file-size distributions. In contrast, when substituting TCP for TFRC (bottom plot in Fig. 6), retaining all other parameter settings including the reduced link capacity, we find, as indicated elsewhere [4], that TCP maintains the LRD structure introduced by heavy-tailed file sizes. This suggests that LRD of aggregate traffic might *not* be insensitive to details in the protocol stack or network configuration, which motivates us to explore the effects of network structure.

### 3.3 Effective of Network Structure: Relative Bandwidth

**Fig. 7:**
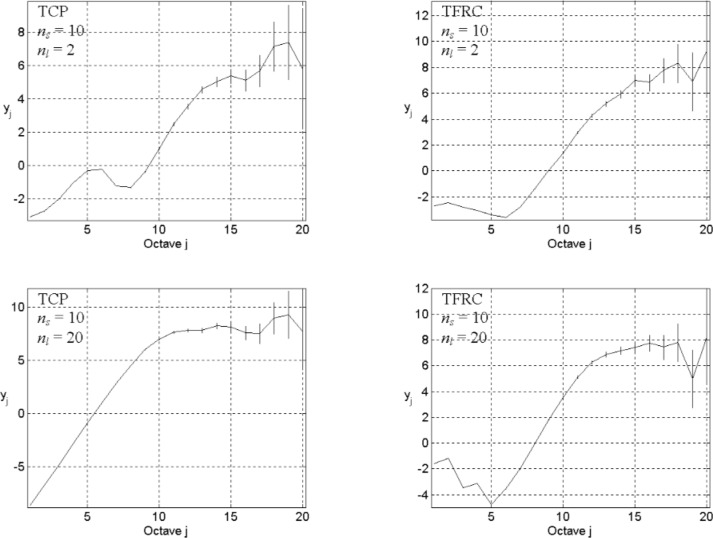
Energy-scale plots for *L* = 3, *n*_s_ = 10, *n*_l_ = 2 (top), or 20 (bottom), the exponential distribution ON/OFF with *λ*_on_ = 200 and *λ*_off_ = 2000, and TCP (left) or TRFC (right) in transport level.

We study the effect of the network structure on traffic dynamics by modulating three parameters: number of sources (*n*_s_), link capacity (*n*_l_), and network size (*L*). We first identify how shrinking or expanding link capacity influences the correlation structure. We set *L* = 3, *n*_s_ = 10, and *n*_l_ = 2 or 20; we use TCP or TFRC as the transport level; we use exponential ON/OFF (*λ*_on_ = 200 and *λ*_off_ = 2000) in the application level. Figure 7 provides the energy-scale plots from the simulation results.

The top row of plots in Fig. 7 depicts correlation structures when *n*_l_ = 2 with either TCP (left) or TFRC (right) used as the transport layer. The bottom row of plots shows correlation structures arising when *n*_l_ = 20 with a transport layer of either TCP (left) or TFRC (right). Recall that, since the basic simulation time step is constant, the finest description of traffic dynamics relies on the selection of *n*_l_. The effects of this fact can be observed in Fig. 7, where the bottom row of plots gives a coarser description for the finest timescale than does the top row of plots.

Examining the correlation structure at large timescales, we find that changing link capacity (*n*_l_) alters the correlation structure. Reducing link capacity tends to strengthen the correlation structure, while expanding link capacity loosens the degree of dependence in the traffic. A similar effect appears through changing *n*_s_.

**Fig. 8:**
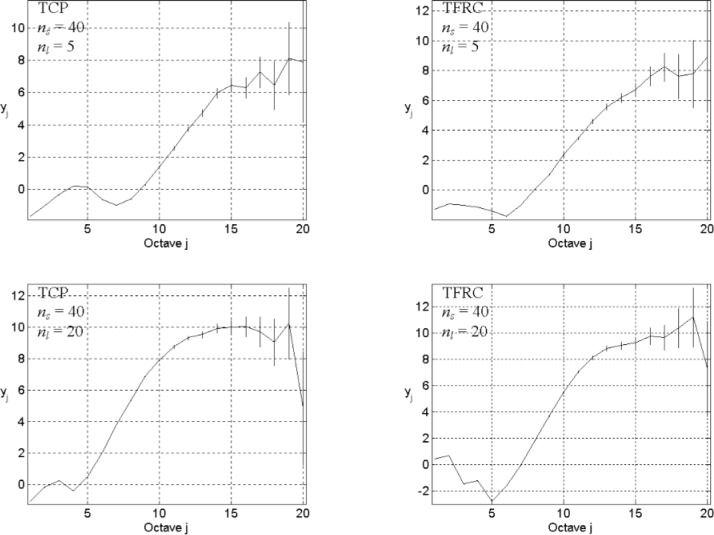
Energy-scale plots for *L* = 3, *n*_s_ = 40, *n*_l_ = 5 (top), or 20 (bottom), the exponential distribution ON/OFF with *λ*_on_ = 200 and *λ*_off_ = 2000, and the TCP (left) or the TRFC (right) in transport level.

Retaining the same network configuration, we set *n*_s_ = 40, and *n*_l_ = 5. In Fig. 8, we show the corresponding energy-scale plots for TCP (top left) and TFRC (top right). Comparing against the top plot in Fig. 3 (TCP with *n*_l_ = 5) and the top plot in Fig. 5 (TFRC with *n*_l_ = 5), we find that increasing *n*_s_ tends to strengthen the correlation structure. The effect is similar to effects from regulating link capacity and varying *λ*_on_/*λ*_off_. To understand this relationship, we also show, in the bottom of Fig. 8, two additional energy-scale plots: one for TCP (bottom left) and one for TFRC (bottom right). The parameters for these plots correspond to the same parameters used in the top row of plots in Fig. 8, except that we increase the link capacity from *n*_l_ = 5 to *n*_l_ = 20. With the increased link capacity, the correlation structures in each plot seem to return to their original shapes, as depicted in the top plots in Fig. 3 (for TCP) and Fig. 5 (for TFRC).

Offered traffic (represented by *λ*_on_, *λ*_off_, and *n*_s_) and shared network capacity combine to act as traffic-shapers, strengthening and loosening correlation structure, which can be offset by congestion-control mechanisms. This view might help to explain why TCP, and its variants, are prone to instabilities when combined with increases in network capacity [24]. Many network protocols and algorithms, including TCP, use measurements of network state to guide future actions. Such methods rely on an implicit assumption that the relevant measures of network state vary sufficiently slowly to have a predictive constancy [25]—i.e., that strong correlation exists at the timescales of interest for the intended controls. Absent such assumptions, the future state of relevant measures would prove very difficult to predict. On the other hand, more surprising interactions might exist in a large network, where the utilization of network capacity could be more influenced by spatial relationships. We investigate this next.

### 3.4 Effect of Network Structure: Network Size

A key property of the Internet is its large size. In July 1998, as reported by Cowie and colleagues [14], the Internet comprised a collection of about 4,000 interconnected routing domains (or autonomous systems). Does network size play a significant role in defining the correlation structure of network traffic?

**Fig. 9:**
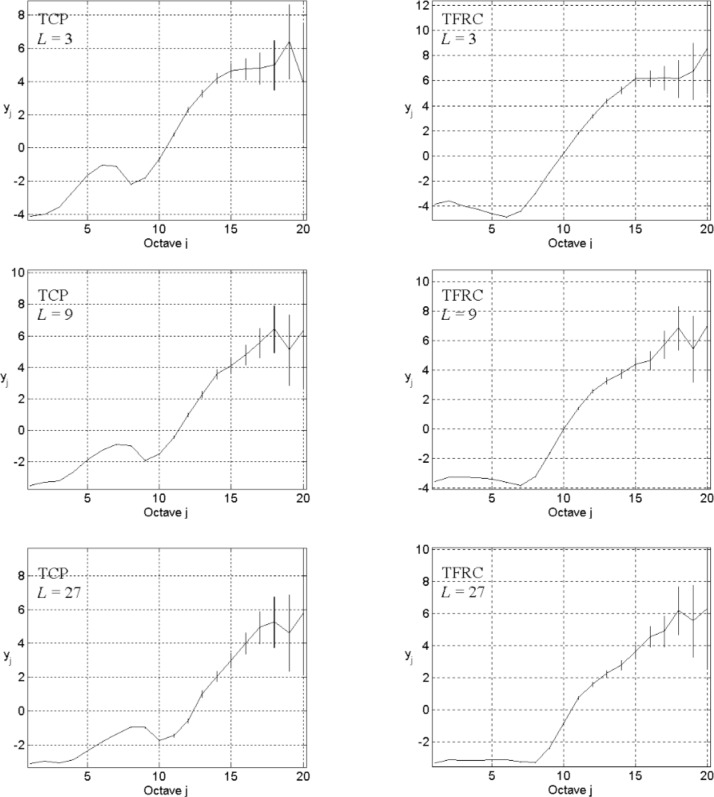
Energy-scale plots for *L* = 3 (top), 9 (middle), and 27 (bottom), *n*_s_ = 5, *n*_l_ = 1, the exponential distribution ON/OFF with *λ*_on_ = 200 and *λ*_off_ = 2000, and TCP (left) or TRFC (right) in transport level.

Even if our model has the potential to answer this question, absent a high-speed parallel computer system, we may still spend a very long time to simulate a network with larger size, capacity, and number of hosts, and to collect sufficient data for wavelet-based analysis. To surmount this obstacle, we chose to investigate a situation where network sources can congest the network backbone, though this is counter to the conventional case where network congestion appears more frequently on network access links. Since our investigation considers comparative rather than absolute results, our limiting assumptions might lead to an informative outcome while reducing computational requirements. We set up our experiments using the following parameters: *n*_s_ = 5, *n*_l_ = 1, and *L* = 3, 9, and 27; TCP or TFRC in transport level; exponential distribution ON/OFF with *λ*_on_ = 200 and *λ*_off_ = 2000 in application level. The energy-scale plots appear in Fig. 9 for TCP (left column) and TFRC (right column), with network size increasing from top (*L* = 3) to bottom (*L* = 27). These plots show that as the network size increases, the flat portion of the curve (indicating exponential decay in the autocorrelation function) diminishes little by little, while the linear portion seems gradually to increase in extent. So it might be reasonable to expect the linear part to extend over some large timescales as the network reaches Internet size, even without high variability in user behavior.

**Fig 10:**
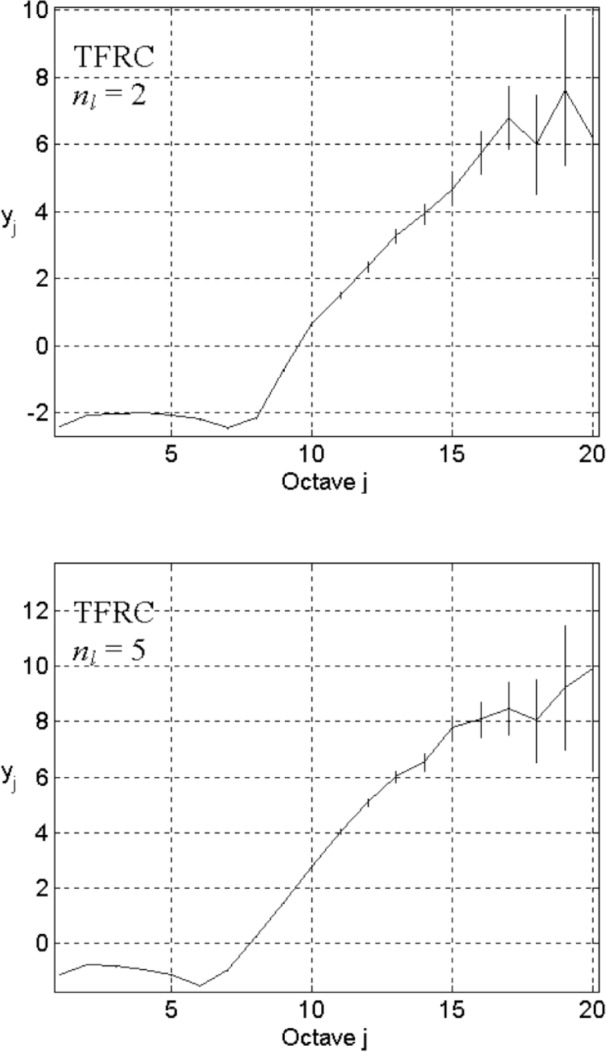
Energy-scale plots for *L* = 15, *n*_s_ = 10, *n*_l_ = 2 (top), 5 (bottom), *λ*_on_ = 200, and *λ*_off_ = 2000, TFRC in transport level, and the exponential distribution ON/OFF.

Will this behavior persist as the Internet evolves toward an increasing amount of streaming traffic, exchanged through rate-based transport protocols? To investigate this question, we assume a network with size *L* = 15 that uses TFRC to transfer data that exhibits low user variability (*λ*_on_ = 200 and *λ*_off_ = 2000). We set *n*_s_ = 10 and *n*_l_ = 2. The top graph in Fig. 10 displays the energy-scale plot from our simulation. Here, our plot shows a similar correlation structure at the medium-to-large timescales (*j* = 10 to 17) as seen in measured Internet traffic. The estimated power-law exponent from the linear part is about 0.819, and thus the Hurst parameter is 0.910. Recall that, as the top plot of Fig. 6 shows, a small network (*L* = 3), even with high user variability, may fail to produce LRD under TFRC. In contrast, LRD appears in this large-scale case (*L* = 15) with the same parameters and without high user variability.

In the bottom graph of Fig. 10, we show the corresponding energy-scale plot using the same parameters, but increasing link capacity to *n*_l_ = 5. The increased link capacity seems to reduce the correlation structure. In this plot (bottom Fig. 10), the slope of the linear part (*j* = 7 to 15) is about 1.197; thus the Hurst parameter does not apply in this case, as its value exceeds one. Figure 10 can be interpreted as saying that the correlation structure of network traffic will probably be controllable by modulating available bandwidth.

## 4. Conclusion

Simulating and understanding large timescale behaviors in large networks present challenges because networks are quite complex and simulation models typically exhibit significant limitations in representational detail, computational tractability, or both. Specifically, most current approaches to network modeling appear unable to produce sufficient data for wavelet-based, long-correlation analysis. We proposed a homogeneous modeling approach that increases computational tractability by raising the abstraction level while retaining many significant details needed to exhibit traffic dynamics as seen in large networks at large timescales. To illustrate the details and the potential of our modeling framework, we examined the effects of traffic sources, transport mechanisms, and network structure on the dynamics of network traffic.

With our model, we simulated the small-scale, middle-scale, and large-scale dynamics of network traffic, which have been identified and discussed extensively by other network researchers. With a wavelet-based data analysis technique, we showed various correlation structures of network traffic, and illustrated variations in energy-scale plots as we changed selected parameters in our model. Regarding the effect of the application level, we verified that high variability in file sizes can result in a strong correlation structure over a wide range of timescales, and that low variability yields linear structure over only a limited range. We also found that high variability in idle time does not necessarily lead to long-range dependence.

Considering the effect of the transport level, we verified that TCP plays an important role in defining traffic characteristics from fine-scale to large-scale. However, we also showed that while both TCP and TFRC can produce strong correlation over a limited range of timescales, their influence on the correlation structure sometimes differs, especially at large timescales, where TFRC does not preserve the LRD structure induced by high user variability. Our results seem to suggest that LRD of aggregate traffic might *not* be insensitive to details in protocol stack or in network configuration.

When investigating the effect of network structure, we found that network configuration can influence traffic dynamics independent of particular transport mechanisms. Specifically, we showed that offered traffic (represented by *λ*_on_, *λ*_off_, and *n*_s_) and shared network capacity combine to act as traffic-shapers, strengthening and loosening correlation structure, which can be offset by congestion-control mechanisms. We gave a comparative simulation result that showed a similar correlation structure to that seen for measured Internet traffic, which might be expected to arise in very large networks, even without high user variability. We suggested a plausible explanation for this result, based on the current understanding of timescale dynamics of network traffic. In large networks, much of the traffic must travel trough multiple domains en route from source to destination. The correlation structure of traffic should arise from the collective effect of all transit flows. Where concurrent connections share one link in a larger network, the longer-distance connections, which need more time for feedback control, must be responsible for the larger timescale correlation structure because connections using either TCP or TFRC can themselves exhibit strong correlation over a more limited range of timescales. Therefore, the spatial span of connections appears to wield significant influence on correlation structure.

While our simulation results justify further investigation of our homogeneous CA modeling approach, cross-verifications with other detailed and simplified models will help to answer whether some aspects of modeling are overly simplified. Using results from such verification studies, we can improve our approach, while retaining its most effective characteristics. We believe that obtaining an in-depth understanding of the spatiotemporal variance of network traffic should be viewed as an important research direction that could lead eventually to a solid methodology for managing large networks.
